# An Evaluation of Personal Health Information Remnants in Second-Hand Personal Computer Disk Drives

**DOI:** 10.2196/jmir.9.3.e24

**Published:** 2007-09-30

**Authors:** Khaled El Emam, Emilio Neri, Elizabeth Jonker

**Affiliations:** ^1^University of Ottawa and Children’s Hospital of Eastern Ontario Research InstituteOttawaCanada; ^2^TrialStat CorporationOttawaCanada; ^3^Children’s Hospital of Eastern Ontario Research InstituteOttawaCanada

**Keywords:** Privacy, confidentiality, security, data disclosure

## Abstract

**Background:**

The public is concerned about the privacy of their health information, especially as more of it is collected, stored, and exchanged electronically. But we do not know the extent of leakage of personal health information (PHI) from data custodians. One form of data leakage is through computer equipment that is sold, donated, lost, or stolen from health care facilities or individuals who work at these facilities. Previous studies have shown that it is possible to get sensitive personal information (PI) from second-hand disk drives. However, there have been no studies investigating the leakage of PHI in this way.

**Objectives:**

The aim of the study was to determine the extent to which PHI can be obtained from second-hand computer disk drives.

**Methods:**

A list of Canadian vendors selling second-hand computer equipment was constructed, and we systematically went through the shuffled list and attempted to purchase used disk drives from the vendors. Sixty functional disk drives were purchased and analyzed for data remnants containing PHI using computer forensic tools.

**Results:**

It was possible to recover PI from 65% (95% CI: 52%-76%) of the drives. In total, 10% (95% CI: 5%-20%) had PHI on people other than the owner(s) of the drive, and 8% (95% CI: 7%-24%) had PHI on the owner(s) of the drive. Some of the PHI included very sensitive mental health information on a large number of people.

**Conclusions:**

There is a strong need for health care data custodians to either encrypt all computers that can hold PHI on their clients or patients, including those used by employees and subcontractors in their homes, or to ensure that their computers are destroyed rather than finding a second life in the used computer market.

## Introduction

The adoption of electronic medical records is growing [1-5]. Concurrently, a majority of patients, and the public in general, are concerned about unauthorized disclosure and use of their personal health information (PHI) in an era of the electronic medical record [6-10].

Concern about privacy has caused some members of the public to not be totally honest with their health care provider [10]. A survey in the United States found that as many as 15% of adults have changed their behavior to protect their privacy [6]. Those behavior changes include going to another doctor, paying out-of-pocket when insured to avoid disclosure, not seeking care to avoid disclosure to an employer, giving inaccurate or incomplete information on medical history, and asking a doctor not to write down the health problem or to record a less serious or embarrassing condition. More than a quarter of teens indicated that they would not seek out health care if they had concerns about the confidentiality of their information [11]. In a survey of US physicians, nearly 87% reported that a patient had asked that information be kept out of their record, and nearly 78% of physicians said that they had withheld information from a patient’s record due to privacy concerns [12]. Similar behaviors have been reported in Canada. A survey estimated that 12% of Canadians have withheld information from a health care provider because of concerns over whom the information might be shared with or how it might be used [13], and an estimated 735000 Canadians decided not to see a health care provider for the same reasons [14]. Such behavior changes can reduce the accuracy of health data [15-18].

Due to inaccurate data, patient safety may be jeopardized: clinicians may make treatment errors [19] or make errors in ordering medications [20]. Furthermore, researchers may underestimate disease prevalence [21], and health system managers may underestimate compliance with standards of care such as vaccination guidelines [22]. Health care organizations may be fined if they report inaccurate data to government agencies [23].

While federal and provincial health care privacy legislation, such as the Health Insurance Portability and Accountability Act in the United States and the Personal Health Information Privacy Act in Ontario, do motivate organizations to properly protect PHI, we do not know the extent to which that has actually been effective in eliminating inadvertent disclosures of PHI.

One relatively easy way to get personal information (PI) about other people is through the disk drives available on the second-hand computer market [24-26]. These computers may have been deliberately resold by their owners (individuals or organizations), donated to good causes (eg, charities or schools) who subsequently sold them, or may have ended up on the second-hand market after they were lost or stolen. In this study we examine the data remnants in second-hand disk drives to determine the extent to which PHI is inadvertently leaking from data custodians. To our knowledge, there have been no studies that have attempted to assess the extent to which PHI can be inappropriately disclosed in this way.

## Methods

One approach to evaluate the extent to which PHI is leaking from data custodians is to count the number of security breaches that are publicized in the media. This, however, has a number of disadvantages: (1) not all security breaches involve PHI (eg, many are of financial data) and media reports may not make the distinction, (2) not all security breaches result in PHI being disclosed (eg, the data was encrypted) and such details usually do not appear in media reports, and (3) only some US states and only one Canadian province have breach notification laws [27]. Therefore, it is plausible that many breaches never get reported in the media.

Consequently, in this study we estimate the prevalence of PI and PHI leaks through second-hand disk drives. Our measure is the proportion (percentage) of second-hand disk drives available on the reseller market with PI and PHI on them. We purchased functional computer disk drives from the second-hand computer equipment reseller market across Canada and examined their contents using digital forensic tools. All nonfunctional drives were returned and replaced.

This study was conducted in the winter of 2006/07. Ethics approval was obtained from the Research Ethics Board at the Children’s Hospital of Eastern Ontario (CHEO) Research Institute.

### Number and Type of Drives

Our focus was on drives that would be used by individual end users (ie, in their desktop machines and laptops). This means that we excluded drives that were used in servers. Hence, we focused on smaller disk drives with a capacity range of 10 GB to 40 GB.

There is no previous research on PHI leaks in second-hand disk drives; therefore, we relied on data remnant studies of PI to determine the number of disk drives needed to estimate the prevalence of PHI [24-26]. We expected the proportion of drives that leak PHI to be smaller than the proportion leaking PI since there is less health information collected and stored electronically compared to other types of PI (eg, financial and legal information). We therefore expected the proportion of drives with PHI to be closer to the lower end of PI, which is 18% [26]. The size of the 95% confidence interval in previous studies that analyzed more than 12 functional drives ranged from ± 9% to ± 11% [26]. We then selected an interval value in the middle: ± 10%, which ensures that the precision of our estimates is within the expected range for this type of study. The minimal number of drives to obtain such a confidence interval is 57. Consequently, we aimed to get data from 60 functional disk drives.

### Identifying Vendors

A comprehensive list of 125 Canadian second-hand computer equipment vendors was identified from telephone directories, contacts and experts in the computer industry, Canadian vendors selling on eBay during the study period, local business directory searches, and a Google search to find “used computer equipment in Canada.” The results were reviewed to form a list of 40 credible potential vendors distributed geographically across the country. We went down the shuffled list and systematically contacted these vendors via telephone and/or email for more information on their inventory. Used disk drives were purchased either in person, over the phone, or the Web, and were picked up personally or shipped to our lab. We limited the maximum number of drives per vendor to 10 so as to ensure a wider distribution of sources.

After contact, some vendors were excluded for a number of reasons:

- They would not sell individual disk drives. Due to cost constraints, we could only purchase stand-alone drives rather than fully configured second-hand computers from which the drives could be physically extracted.
- Some vendors in rural areas did not want to ship the equipment across the country or did not accept payments by phone or over the Internet.
- Some vendors did not have disk drives within the stipulated size range in stock at the time of the study.

We were able to purchase equipment from 12 different vendors (multiple sites for retail chains were counted as multiple vendors).

### Data Recovery

Functioning drives can be classified as blank, recoverable, or securely wiped. Blank drives were readable but there was no data on them at all, current or deleted. Data on drives that have been formatted or repartitioned can be quite easily recovered [24]. Files that are deleted are also recoverable since a delete does not actually remove the data from the drive but only removes the entry from the file directory. We used a commercial software package called Recover My Files (GetData Pty Ltd, Hurstville, NSW, Australia, version 3.98, build 5282) to retrieve the data from the drives and recover the files that have been deleted [28]. The same tool was used to recover data from formatted and repartitioned disk drives. Further information about data recovery is included in the Multimedia Appendix.

It is possible to use tools that implement a specific secure delete algorithm that ensures that the data cannot be recovered. The DOD 5220.22-M standard is a US Department of Defense standard providing specifications for clearing and sanitizing electronic data storage devices [29-31]. There are some commercial and freely available tools that implement that standard (eg, see [32]). It is not possible to extract the data from such drives. For drives that were not blank and that were not recoverable, we used a hexadecimal editor to read the patterns of data on the disk. Disks that have been wiped using this approach either have a single character (usually a zero) written to the disk or have a characteristic pattern of alternate ones and zeros followed by a random character written to the disk.

### Data Coding and Analysis

All data from the recovered drives were stored on DVDs. A search of the files on each DVD was performed in order to isolate files that may have contained PI and PHI. The DVDs were searched for Microsoft Word documents, Excel documents, PowerPoint documents, Outlook files, Access database files, Adobe (PDF) documents, and text files. All discovered files were manually reviewed and a summary of the discovered PI and PHI was completed for each disk. An attempt was also made to identify the owner(s) of each disk drive from the information it contained.

PI was defined as identifying information (eg, name, address, social insurance number) about an individual or individuals *plus* other sensitive but nonmedical information (eg, financial information, personal correspondence, divorce documents, legal records). PHI was defined as identifying information about an individual or individuals *plus* any sensitive physical or mental health information. By this definition, if a drive only had a list of names and addresses but no sensitive information associated with them, then such data would not be considered PI or PHI. This definition is somewhat conservative because one can argue that a list of names and addresses suggests that all of these individuals were associated in some way; hence, association information would be revealed. Therefore, our results would be considered a lower bound on the prevalence of PI and PHI in the second-hand drives we analyzed.

Four ratings were made for each drive depending on the type of information it contained: (1) PI about the owner, (2) PI about other individuals apart from the owner, (3) PHI about the owner, and (4) PHI about other individuals apart from the owner. If a drive was clearly owned by multiple people (eg, members of a family), then they were all considered the owner in our coding. This means that, for example, if PI existed on any one of them, then the drive was considered to contain PI on the owner.

When considering whether information about the owner was really PI or not, we needed to decide what to do about *work products*. A work product is the output of an individual’s professional or employment responsibilities. For example, a physician’s prescription record would be considered a work product of the physician, irrespective of whether the patient is identifiable or not. The Federal Privacy Commissioner of Canada does not consider work products to be PI [33]. However, the European Commission had a different interpretation and considers information on and *relating to* an individual regardless of the position or capacity of the individual, such as a prescription record written by a physician, a communication of PI [34]. Given the uncertainty across jurisdictions, we treated information deemed to be work products as PI in one analysis and not PI in another analysis and present both sets of results.

Two independent individuals rated the drives. The first rater analyzed all of the 60 drives. The second rater analyzed a subset of the drives to ensure that the coding was reliable. Where there was disagreement, the two raters met to discuss their rationale and reach a consensus rating.

To determine how many drives the second rater needed to analyze, we performed a power analysis for using the Kappa statistic [35]. Given that there are no precedents for the interrater reliability of data extraction from second-hand drives, we relied on generally accepted benchmarks for Kappa values. Hartmann notes that Kappa values should exceed 0.6 [36]. Landis and Koch provide a more general benchmark, where values between 0.4 and 0.6 are considered moderate agreement [37]. A similar benchmark is provided by Altman [38]. Fleiss suggests that values between 0.4 and 0.75 represent intermediate to good agreement [39]. To err on the conservative side, we assumed that our value of Kappa would be at least 0.5, which would be considered a moderate level of agreement according to the above benchmarks. At that level of agreement, and 80% power to reject a null hypothesis comparing Kappa to agreement by chance, the second rater needed to code only 32 drives [40,41].

The final results are presented in terms of the percentage of disk drives containing PI and PHI on owners and other individuals, with the 95% confidence interval [42]. Interrater agreement is presented in terms of the Kappa statistic and its 95% confidence interval.

### Special Protocols

Three special protocols were put in place for this study:

1. Some disk drives were expected to contain inappropriate/obscene material (eg, pornography). We therefore did not explicitly look through image files (file extensions .gif, .jpg, .psd, .tif, and .bmp). Also, one member of our research team initially screened the drives for files and directories with suggestive names and flagged these particular drives as potentially containing such materials. No searching of files with suggestive names was done on flagged drives.
2. If any illegal materials were discovered (eg, child pornography), that information was to be passed on to the police.
3. If there were cases of disclosure of particularly sensitive PI or PHI for a large number of individuals, then they were to be reported to the appropriate (federal or provincial) privacy commissioner for follow-up.

## Results

All of the 60 drives were from personal computers and ran the Windows operating system. Repartitioning and formatting are two common approaches for manipulating the drives. However, as noted earlier, much data can be recovered despite these efforts. There were 35 drives that were repartitioned or formatted, and 5 that had had nothing done to them (all data were readily available). Therefore, data was potentially recoverable from 67% (95% CI: 54%-77%) of the drives.

A significant percentage of drives (28%; 95% CI: 19%-41%) were wiped using the DOD 5220.22-M standard. Three of the drives (5%) were completely blank and there had not been any data written to them. Five of the drives had pornographic files on them. Two of the drives were referred to the provincial privacy commissioner’s office due to the sensitivity of the data that was found. No cases were referred to the police.

We were able to retrieve data from 39 drives (one of the repartitioned drives had no data on it). This represents 65% (95% CI: 52%-76%) of the total. Overall, we extracted 57 DVDs of data from the 39 drives.

**Table 1:** Contingency table with marginal totals and percentages showing the status of purchased drives distributed by province of purchase*

Province	Repartitioned	Formatted	DoD 5220.22-M Standard	Data Readily Available	Blank	Total
Ontario	19	11	5	4	3	42 (70%)
Quebec		5				5 (8%)
Alberta			12			12 (20%)
British Columbia				1		1 (2%)
Total	19 (32%)	16 (27%)	17 (28%)	5 (8%)	3 (5%)	60 (100%)

^*^For store chains, we considered the location of the specific store that we purchased from. The actual owners of the disk drives may be located in a different province or country. Four of the drives bought from Ontario belonged to US-based entities: 2 of them were state government departments, 1 was a municipal department, and 1 belonged to an individual.

There were 7 vendors in Ontario, 1 in Quebec, 3 in Alberta, and 1 in British Columbia. The distribution of drives by province is shown in Table 1. There are relatively more drives purchased from Ontario. Data were extracted from the drives from 6 of the Ontario vendors. All of the drives purchased from Alberta had been securely cleared such that no data were recoverable.

After our data analysis, we contacted the 3 Alberta vendors to understand why they all had used secure methods for deleting data on the drives they resell. They all stated that they had internal standard operating procedures for doing secure deletes on the second-hand disk drives that they sell because they do not want to get involved in any data breaches.

We were able to determine the address of the drive owner for 26 of the 39 drives with recoverable data. All of the 26 disk drives came from urban areas (using the Canada Post and the Canadian Medical Association definitions of a rural postal code, which is only one of multiple possible definitions [43]). The owners for 22 of these 26 drives were in the same province as the vendor. The other 4 were US-owned but were sold by Ontario vendors.

**Table 2:** Claims made by the vendors of the drives from which we were able to extract data

Vendor Statement About Wiping Drives	Count
“Like new condition”	1
Verbally stated that the drives were formatted	1
“Recertified to factory settings”	1
None	5

The 8 vendors from whom we bought drives that had data on them did not actually make any claims that the data would be removed in a secure way (see Table 2). Therefore, they were not in breach of any agreements that they had made.

**Table 3:** Percentage of drives with recoverable files and percentage of total drives with available personal data

	Owner PI (A)^*^	Owner PI (B)^*^	Other PI	Owner PHI	Other PHI
Percentage of Recovered (95% CI)	72% (28/39) (56%-83%)	62% (24/39) (46%-75%)	56% (22/39) (41%-71%)	13% (5/39) (6%-27%)	15% (6/39) (7%-28%)
Percentage of Total (95% CI)	47% (28/60) (35%-59%)	40% (24/60) (29%-53%)	37% (22/60) (26%-49%)	8% (5/60) (7%-24%)	10% (6/60) (5%-20%)
Kappa^†^ (95% CI)	0.8 (0.6-1)	0.6 (0.33-0.88)	0.78 (0.54-1)	0.76 (0.45-1)	0.795 (0.52-1)

^*^(A) indicates that work products were considered as PI, and (B) indicates that work products were not considered as PI.

^†^Interrater agreement Kappa scores and their 95% confidence intervals.

A summary of the type of data that was uncovered in these drives is shown in Table 3. All Kappa scores were above our threshold of moderate agreement. The vast majority of drives with data had PI about their owners (close to half of all drives) and about others. Examples of PI found on the disk drives included:

- personal budgets, salary information, tax returns and completed tax filing forms
- letters regarding personal relationships
- information on life insurance policies and inheritances
- payroll records of employees, including addresses, dates of birth, and social insurance numbers
- email correspondence regarding employees and their actions
- police record checks
- divorce documents

A considerable percentage of the total drives had PHI about the owners (8%) and other people apart from the owner (10%). The vast majority of that information was in correspondence (eg, Word documents, PDF documents, and email). Examples of PHI found on the disk drives included:

- psychological assessments of adults and children, correspondence related to custody cases involving children, affidavits, and social history of abuse victims
- medical certificates
- letters regarding alcohol addictions of other individuals (not the owners of the drive)
- reports from a registered nurse about other individuals’ health problems, cases of abuse, children’s health, and medication lists
- correspondence regarding the placement of adults and children in long-term care facilities

The 6 disk drives with PHI about other people came from 3 different vendors; half of them were for personal use and the other half belonged to organizations.

## Discussion

We found that PI, including PHI, was recoverable from 65% of the drives we purchased; 8% of the disk drives had PHI of the owners, and 10% of all drives had PHI on other people. Half of the latter set came from organizations that were directly entrusted with that information and the other half from people working for such organizations (eg, a nurse who took some information home to work on it).

Approximately 8% of personal computers in use worldwide are second-hand machines [44], out of a total installed base of 980 million in 2007 [45]. To the extent that our findings are generalizable, an 8%-10% prevalence of PHI in second-hand disk drives in use paints a disturbing picture about the inappropriate disclosure of PHI. The second-hand computer market is expected to grow significantly in the next few years [46,47] and, with it, the opportunity for further such disclosure of PHI.

In a previous data remnants study done in the United States [24], 158 drives were bought. Of these, 129 were successfully imaged. Approximately 9% were wiped. It was possible to extract data from many of the remaining drives (38%). A similar international study provided the results presented here in Table 4. PI was recoverable from 60% of North American drives, although the sample size was quite small. Based on data from the United Kingdom and Australia, the range of drives with PI is 18% to 49%. While our findings on PI are consistent with previous studies, giving them some face validity, previous studies did not consider PHI.

**Table 4:** Summary of findings from an international data remnants study [26]

	UK and Australia (2005)	UK (2006)	Australia (2006)	Germany (2006)	North America (2006)
Total Drives	116	200	53	40	24
Faulty Drives	13 (11%)	87 (43%)	3 (6%)	30 (75%)	12 (50%)
Wiped^*^	17 (16%)	55 (49%)	18 (36%)	4 (40%)	1 (8%)
Had PI^*^	51 (49%)	35 (31%)	9 (18%)	3 (30%)	7 (60%)

^*^The percentage of these disk drives that were not faulty.

### Prevalence of PHI

Our results indicate that not as much health information is leaking as other types of information, such as financial and legal information. Why is relatively less PHI available electronically on these drives?

In Canada, the use of computers and the Internet is quite common. The majority of the population has access to a home computer [48], and most citizens have access to the Internet [49,50]. However, this does not mean that they have easy electronic access to their own PHI.

There are a number of ways that individuals can get electronic access to their own health data. For example, individuals may request their medical records from the institution that provided them with care. In practice, very few hospitals provide medical records electronically or make them accessible [51]. Another study found that a very small percentage of members of an integrated delivery system used eHealth services when provided to them [52].

It is more likely that PHI will exist in correspondence, such as email. The proportion of US Internet users who reported communicating over the Internet with their health care provider in 2005 was 10% [53]; a European survey found that 4% have approached their family doctor over the Internet, and about 7% of email users in the United States exchange emails with physicians or health professionals [54]. The proportion of physicians who report communicating by email with their patients varies from 3.6% to 24% [55-57]. About a quarter of patients correspond via email with family members [50]. PHI may also be exchanged electronically with peers [58]. This is consistent with our findings in that most of the PHI we found was in correspondence rather than in electronic medical records or database files.

One would expect that as electronic medical records become more widely deployed, more PHI will be available to patients electronically and hence the risk of inappropriate disclosure of PHI will increase over time. The disclosure risk is highest with care and service providers who would have extensive electronic correspondence with and documentation on many patients and clients on their work and personal computers.

### Practices for Securing Data on Disk Drives

There is clearly a need for organizations and individuals, and certainly in Ontario and to some extent Quebec, to take actions to reduce the risk of personal data leaking from second-hand disk drives. A disk can leave its custodian in three ways: it is destroyed, it is given away, or it is lost or stolen.

The safest way to dispose of a disk drive is to properly destroy it. Approximately 38% of all used personal computers, including their drives, are destroyed [59]. While there are a large number of techniques that an individual or organization can potentially employ to destroy equipment [60], many of them require specialized equipment or resources and it is therefore not practical for most users to do it themselves. However, destruction of equipment can be outsourced to specialized vendors.

If equipment will be donated or resold, the risks of PHI leaks remain high. Donated equipment may end up in foreign second-hand markets, as demonstrated by a recent case of British computers ending up in Africa [61]. Approximately 6% of second-hand computers are exported [59]. If not exported, local resellers will not necessarily wipe data from the drives they acquire [62].

There are three general approaches that can be pursued to protect data on equipment before it is given away: de-identification of data, encryption of data, and the use of secure delete technology. Such approaches should be applied on all computers that will hold PHI, including the personal computers of staff and contractors who may take data home to work off-site.

Any PHI on a disk drive ought to be de-identified at the earliest opportunity. De-identification ensures that the risk of finding out the identity of the individuals about whom the data pertains is low [63]. This means removing or masking directly identifying data and applying other anonymization techniques to protect indirectly identifying data [64]. However, there will be many cases when data need to be identifiable to be useful. Hence, additional techniques would also need to be used.

Another way to protect data is by using encryption technology. Encryption can be used to create specific virtual drives, and all sensitive information can be stored on the virtual drives. Unless the password used is weak or the encryption algorithm is compromised, it would be extremely difficult to extract the information. However, this is generally not enough. Many programs will store their data, temporary files, cached files, backup files, and registry values outside the encrypted virtual drive. Quite a significant amount of information can be left in these files. Most users would not know to change the settings of their applications to only use the encrypted disk drive, and sometimes that option is not available. Therefore, if one really wants to protect data, this would probably not be the best approach unless one possesses a great deal of technical expertise (to change the setting of the applications to force them to use the encrypted drive).

The best encryption technology to use is whole disk encryption [65] that is invoked before the operating system, during system boot, starts to operate. This ensures that all data on the drive (temporary, backup, and data) are encrypted. Fortunately, this type of technology is becoming more generally available in common operating systems and hardware. Therefore, one would expect that, in the next few years it will be much more widely deployed and will significantly reduce the risks we have identified. Specific stand-alone products are listed by the Privacy Commissioner of Ontario in a fact sheet [65].

The second technology one can use is secure delete. This allows one to delete all of the data on the drive so that they are not recoverable (such as when using the DoD 5220.22-M standard). Secure delete by itself, however, is not enough. One needs to perform a more general disk wipe. Software for wiping disks usually performs a secure delete as well as removing all of the temporary, backup, and cached files from the system.

A recent study noted that commercial software for wiping disks tends to be quite unreliable [66]. In one case, the software did not even attempt a secure delete because of a software bug. The difficulty with wiping software is that the program needs to determine where each application keeps its information. This is difficult to do for a very large number of applications that change often. It has been argued that because the market for privacy tools is small (and hence the vendors have limited resources), such vendors will not be able to keep up-to-date with the application and operating system changes [66]. Therefore, while the use of wiping software is reassuring, it may not actually be sufficient to protect personal data on disk drives.

Even if an organization does not resell or donate its equipment, theft and loss are real risks. For instance, a recent survey noted that 47% of organizations reported theft of a laptop or mobile computing device [67]. Some recent health care examples: (1) the theft of 2 laptop computers containing the names, birth dates, addresses, PHI, and insurance information for 3000-4000 patients was reported after a break-in at a rehabilitation clinic [68], (2) a laptop computer that contained 51 assessment reports was stolen from the car of a psycho-educational consultant working for the school board [69], and (3) a laptop computer containing data on 2900 patients participating in clinical trials was stolen from a researcher’s car [70]. All of the techniques described above that are used to protect data when equipment is donated or sold should therefore be considered even if there is no intention to part with the equipment. One cannot control loss or theft events.

In summary then, it is best to properly destroy equipment when it is no longer in use. Even if that is not possible or desirable, it is still advisable to have full drive encryption to be activated as soon as the computers are purchased. With full drive encryption, there is minimal risk (unless the passwords used are weak) if the disk drive is given away, lost, or stolen at a later date.

### Limitations

In this study we only examined one source of leakage of PHI from data custodians. However, our results indicate that this is an important source of very sensitive PHI. As more information is stored and exchanged electronically, the risks from such leakage are bound to only increase unless current practices change.

We explicitly limited the study to Canadian vendors since this geographic location has not been studied before and because Canada has strong federal privacy laws. Hence, one would have expected that the ability to find PHI would be quite low—which was not the case.

The representativeness of the 60 drives of all drives in the second-hand market is a concern. We would argue that this sample underestimates the problem for a number of reasons. First, we excluded large disk drives, which eliminated data from servers. Servers would potentially contain large databases of PI and/or PHI. Second, some vendors (eg, those in rural locations) became suspicious of our motives for purchasing disk drives (“Why is someone from Ottawa buying a single drive from rural Alberta?”) and therefore refused to sell. We suspect that vendors with drives containing un-wiped PI and/or PHI were less likely to sell us the equipment. Therefore, our results should be considered a lower bound on the extent to which PI and PHI leaks through second-hand drives.

We did not specifically seek disk drives with health information on them. Had we done so, the PHI proportions would likely have been higher. However, that would not have provided a realistic assessment of the risk. Second-hand equipment vendors do not specialize by domain (ie, there were no vendors that specialize in selling only used equipment from health care facilities). Had we specifically requested equipment from health care institutions, it may have sounded like a suspicious request and dissuaded the vendors from completing the transaction.

## Multimedia Appendix

Details of the data recovery process
					

## Abbreviations

PHI: personal health information
PI: personal information
