# Supplementary material for: An Evaluation of Personal Health Information Remnants in Second-Hand Personal Computer Disk Drives
Source: J Med Internet Res. 2007 Sep 30;9(3):e24. doi: 10.2196/jmir.9.3.e24 (PMC2047285; doi:10.2196/jmir.9.3.e24)
Supplement: Supplementary file 1 [file jmir_v9i3e24_app1.pdf]

Multimedia Appendix

Data Recovery

Data recovery is a process by which an individual may retrieve data from a previously deleted data storage medium. This data may be a file or set of files that have been simply deleted from a system or an entire volume of data that has been removed from a magnetic disk storage device via the formatting or repartitioning of the entire disk. A general misconception exists among many users of computer systems that once a file has been deleted, the data is forever destroyed. This is not necessarily the case.

In order to properly understand methods for data recovery, one must understand the fundamental structures of file systems utilized in magnetic disk storage devices. In essence, the structure of a magnetic disk storage device file system can be viewed as being composed of the following major sections (from the beginning to the end of the structure):

Boot Sector
Reserved Sectors
File Allocation Table #1
File Allocation Table # 2
DATA (Containing the Root Directory)

The first section of the file system is the Boot Sector. This contains the executable code that is passed to the central processing unit by the BIOS at boot time (after POST has been completed). The Boot Sector also contains information about the physical structure of the disk and handles the data access at boot time. The executable code within the Boot Sector then launches the Operating System contained in the DATA section of the disk.
The File Allocation Tables on a disk contain information that tracks the allocation of sectors as well as the grouping of all the clusters on a disk. As an example, this would have an entry stating that a cluster is located at a specified physical location of a disk. A cluster can also be referred to as an allocation unit and is the smallest unit of disk space that can be allocated to a single file. Two File Allocation Tables exist in order to provide redundancy on a disk should one fail.

A Root Directory or Root Folder (also referred to as the System Area) is located within the DATA area of a disk and is referenced within the File Allocation Table. This Root Folder contains a record of the mapping of files to clusters on the hard disk. These file clusters are contained in the remaining areas of the magnetic disk.

When a file or folder is deleted from the magnetic disk storage device by the operating system, the first character of the file or directory name is changed to Sigma - ASCII symbol 229 (0xE5) and all pointers for this file are removed from the Root Folder leaving the data in the clusters to be overwritten by the operating system at some later date. The reason why this has been done in operating systems is to increase their speed and efficiency when working with files. When a magnetic disk storage device is formatted, the Root Directory is purged, but the data remains on the drive as per a file deletion. In the case of repartitioning, the File Allocation Tables and the Root Directory are purged, but all data still remains within the clusters of the disk until it is overwritten.

To recover data, all one would have to do is parse through the data in each cluster of a DATA section in order to reconstruct missing data. In the case of simple file deletion, all one would have to do is parse through the DATA clusters looking for clusters containing the Sigma character. Once a cluster is found that contains a Sigma character, one can inspect the data within the cluster. Information encoded within this data region can be used to recreate the cluster chain (the location of all the parts of the file across

the disk). Once a cluster chain is determined, the file can be reconstructed on another storage medium.

If a partition has been deleted, the method is much easier for data recovery. In this scenario, all that one would have to do is iterate through each cluster in order to extract the cluster chain and then reconstruct the files on another storage media. This same method can also be utilized for formatted magnetic disk storage media.

This process can easily be automated through data recovery tools. Several commercial as well as freeware software packages are available.
